# THE BENEFITS OF POSITIVE SOLITUDE IN LATE LIFE

**DOI:** 10.1093/geroni/igad104.1055

**Published:** 2023-12-21

**Authors:** Yuval Palgi, Ehud Bodner, Dikla Segel-Karpas

## Abstract

Positive solitude is the capacity to choose spending time by yourself in a positive manner. This session provides a wide glance on the advantage of this capacity at the second half of life. The session will try to present new theoretical perspectives regarding this capacity. The lectures will give an opportunity to follow the directions in which research in this new field is being developed. The first lecture by Palgi will describe the theoretical background for the study of positive solitude, and will provide new findings from the positive solitude scale that was lately develop by the authors. The second presentation by Bodner will describe how emotional regulation through music moderates the relationship between mindfulness and positive solitude. The third lecture by Zambrano Garza is contributing to the understanding of solitude in the interpersonal domain. Based on dyadic diary study, demonstrates that more voluntary solitude was associated with more positive affect of the partner and more negative solitude was related to more negative affect of partner. The forth lecture by Segel-Karpas will focus on the contribution of positive solitude and loneliness on negative aspects of mental health. Finally, the fifth presentation, by Jennifer Lay, will show use natural language processing to identify solitude experiences from older adults’ reports. We will conclude the session by discussing future research directives for the implementation of positive solitude in the field of gerontology, such as the development of interventions that may enhance the tendency for positive solitude in old age.

